# Machine Learning-Based Lung Cancer Classification Using Blood-Derived Microbial DNA: A Comparative Analysis of Taxonomic Profiling Strategies

**DOI:** 10.3390/diagnostics16071079

**Published:** 2026-04-02

**Authors:** Chul-Jun Goh, Jiwoo Park, Yoonhee Kim, Dabin Park, Jinkyoung Kim, Sun Jae Kwon, Min-Jeong Kim, Min-Seob Lee

**Affiliations:** 1 Eone-Diagnomics Genome Center, Inc., 143, Gaetbeol-ro, Yeonsu-gu, Incheon 21999, Republic of Korea; cj.ko@edgc.com (C.-J.G.); jw.park@edgc.com (J.P.); yh.kim@edgc.com (Y.K.); dabin.park@edgc.com (D.P.); jk.kim@edgc.com (J.K.); minjeong.kim@edgc.com (M.-J.K.); 2 AccuGene, Inc., 73, Gaetbeol-ro, Yeonsu-gu, Incheon 21999, Republic of Korea; 3 Diagnomics, Inc., 5050 Murphy Canyon Road, Suite 150, San Diego, CA 92123, USA

**Keywords:** circulating cell-free microbial DNA, lung cancer, machine learning, taxonomic profiling, bioinformatics pipeline, liquid biopsy, low-biomass microbiome

## Abstract

**Background**: Blood-derived circulating cell-free microbial DNA (cfmDNA) has emerged as a potential non-invasive biomarker source for cancer detection. However, low biomass and high susceptibility to analytical variability raise concerns regarding the stability and interpretability of inferred microbial signatures. This study aimed to evaluate how different taxonomic profiling strategies influence downstream machine learning-based classification and feature interpretation in lung cancer. **Methods**: cfDNA sequencing data from 168 individuals (80 lung cancer patients and 88 non-cancer controls) were analyzed using two taxonomic profiling workflows: a Bracken-based abundance estimation approach and a BLAST-refined alignment-based strategy. Microbial profiles derived from each pipeline were evaluated using supervised machine learning models within a nested cross-validation framework. Feature stability and fold-change trends were compared across profiling strategies. **Results**: A Random Forest model achieved robust classification performance under both workflows (AUC 0.852 for Bracken-derived data and 0.906 for BLAST-derived data). However, substantial pipeline-dependent variation was observed in feature selection patterns and quantitative fold-change directionality. Although 13 genera were consistently selected across cross-validation folds in both workflows, the magnitude and direction of abundance differences were not uniformly concordant. **Conclusions**: Blood-derived microbial DNA profiles can support machine learning-based lung cancer classification; however, feature-level interpretation remains sensitive to taxonomic assignment strategy. These findings underscore the importance of pipeline-aware interpretation and methodological transparency in low-biomass blood microbiome research.

## 1. Introduction

Cell-free DNA (cfDNA) analysis has become a central component of non-invasive molecular diagnostics, particularly in oncology, where circulating tumor DNA enables real-time monitoring of tumor burden and genomic alterations. Beyond host-derived cfDNA, circulating cell-free microbial DNA (cfmDNA) has been detected in blood and has emerged as a potential source of non-invasive biomarkers for infectious diseases and cancer [1,2,3,4,5,6].

Circulating microbial DNA is thought to originate from multiple sources, including translocation of microbial components from barrier sites such as the gut, release from tumor-associated microbiota, and microbial DNA fragments derived from infections or dysbiotic microbial communities [7,8]. In cancer, microbiome dysbiosis and increased epithelial permeability may facilitate the entry of microbial DNA into the bloodstream, where it can reflect disease-associated microbial signatures [9,10]. Accordingly, circulating microbial profiles have been proposed as a novel class of biomarkers with potential utility in cancer detection and classification. In the context of lung cancer, there remains a strong clinical need for accurate and non-invasive biomarkers that can support early detection and improve diagnostic stratification, as conventional diagnostic approaches are often limited by invasiveness or insufficient molecular resolution [11].

Recent studies have demonstrated that disease-associated microbial signatures can be detected in blood across multiple cancer types, including lung cancer [12,13,14,15]. Parallel investigations have consistently identified tumor-associated microbiome alterations in colorectal, gastric, and breast cancers [16,17,18,19,20], supporting the broader concept that cancer-related microbial dysbiosis extends beyond local tumor environments and may be reflected systemically.

Notably, emerging evidence indicates that circulating microbial DNA profiles can discriminate lung cancer patients from non-cancer controls and may enable risk stratification across independent cohorts [9,10]. Collectively, these findings highlight the growing potential of blood-derived microbiome profiling as a non-invasive approach for cancer detection.

However, interpretation of microbial signals in blood presents distinct analytical challenges. Circulating microbial DNA is typically characterized by extremely low biomass, susceptibility to environmental and reagent contamination, and strong dependence on bioinformatics processing choices [7,8,9,21]. In a low-biomass context, technical variation may meaningfully influence inferred taxonomic composition and downstream biological conclusions.

Despite these limitations, taxonomic profiling outputs are frequently treated as stable representations of the blood microbiome, with primary emphasis placed on classification performance or the identification of candidate taxa [12]. However, systematic evaluation of how bioinformatics pipelines influence feature selection stability, quantitative abundance patterns, and machine learning-based interpretation remains limited. This represents a critical gap, particularly in cfDNA-derived datasets, where analytical choices can substantially alter inferred microbial composition and, consequently, biological conclusions.

**Figure 1:**
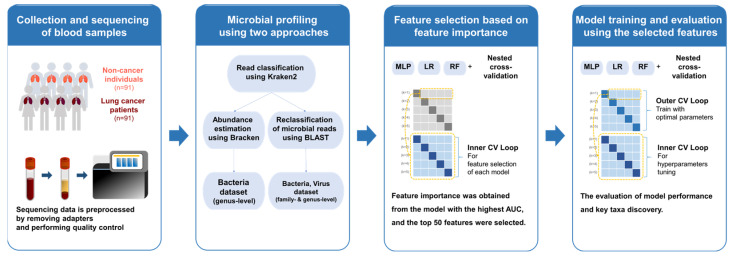
Schematic overview of the analytical pipeline.

In this study, we present a comparative bioinformatics framework to evaluate cfDNA-derived microbial profiles for lung cancer classification. By applying two widely used taxonomic profiling strategies—a Bracken-based abundance estimation workflow and a BLAST-refined alignment-based approach—to the same sequencing dataset, we directly assess how analytical stringency influences classification performance and microbial feature interpretation (Figure 1).

Rather than proposing individual taxa as definitive biomarkers, we focus on evaluating the robustness and pipeline sensitivity of microbial signatures in low-biomass cfDNA data. Through this approach, we aim to establish a more cautious and methodologically transparent framework for blood-based microbiome research. From a clinical perspective, such analytical robustness is essential for translating cfDNA-derived microbial signatures into reliable diagnostic tools, as the lack of standardized and reproducible pipelines may limit clinical applicability despite strong classification performance.

## 2. Materials and Methods

### 2.1. Sample Preparation

Blood samples were initially collected from a total of 182 individuals. After quality control and read-level filtering (described in Section 2.2), 14 samples were excluded due to insufficient sequencing quality, resulting in a final cohort of 168 individuals, including 80 lung cancer patients and 88 non-cancer controls used for downstream analyses. Clinical characteristics of the study cohort, including sex distribution, cancer stage, and histological subtype, are summarized in Table 1.

Each individual provided two 10 mL blood samples collected using Cell-Free DNA blood collection tubes (EDGC, Incheon, Republic of Korea). Plasma was isolated according to standard protocols, and cfDNA was extracted from 4 mL of plasma using the Chemagic 360 system (PerkinElmer, Waltham, MA, USA) with the Chemagic Circulating NA 4K 360 H24 protocol. cfDNA concentration was quantified using a Qubit 2.0 fluorometer with the dsDNA High Sensitivity Assay Kit (Life Technologies, Carlsbad, CA, USA).

Library preparation was performed using the TruSeq DNA HT sample preparation kit (Illumina, San Diego, CA, USA), and sequencing was conducted on the NextSeq 550Dx platform (Illumina, San Diego, CA, USA) using the NextSeq High Output Kit (Illumina, San Diego, CA, USA). Sequencing was performed using paired-end reads (2 × 38 bp) with an average sequencing depth of approximately 0.5× per sample.

No-template or reagent-only negative controls were not included in this study, as the analysis was conducted retrospectively using pre-existing sequencing data. Given the low-biomass nature of circulating microbial DNA, this represents an inherent limitation and should be considered when interpreting microbial signals. To mitigate potential contamination effects, downstream analyses incorporated computational filtering strategies based on previously reported contaminant taxa and cross-validation-based procedures to reduce systematic bias [7,8,21].

### 2.2. Data Preprocessing

Adapter trimming and quality filtering were performed to minimize the impact of sequencing artifacts, which can disproportionately affect taxonomic classification in low-biomass cfDNA samples. Adapter sequences were removed using Trimmomatic (ver.0.39) [22], and read quality was assessed using FastQC (ver. 0.11.5) [23]. Bases with a Phred quality score below Q30 were trimmed, and reads shorter than 20 bp or unpaired reads were discarded. The use of a stringent Q30 threshold was intended to reduce spurious alignments arising from low-quality bases, which is particularly critical for downstream microbial classification in short-read cfDNA data.

After quality filtering, samples with insufficient sequencing quality were excluded from further analysis. Specifically, samples with low total read counts or abnormal quality distributions were removed, resulting in the exclusion of 14 samples. The final dataset consisted of 168 samples (80 lung cancer patients and 88 non-cancer controls) used for downstream analyses.

On average, approximately 60.1 million reads were obtained per sample after quality filtering, with comparable sequencing depth between normal (59.5 million) and lung cancer samples (60.8 million). The majority of reads were classified as human (mean 97.34%), while a small fraction was assigned to microbial taxa (mean 0.56%), consistent with the low-biomass nature of circulating microbial DNA. The proportion of microbial reads was similar between normal (0.58%) and lung cancer samples (0.53%).

Following quality control, processed reads were subjected to downstream taxonomic classification and host read filtering as described in Section 2.3.

### 2.3. Microbial Profiling

Kraken2 [24] was used for initial read classification due to its computational efficiency and ability to rapidly separate host-derived and microbial reads, which is advantageous for large cfDNA datasets. Kraken2 was run with the parameters “--paired--confidence 0.15”. A relatively permissive confidence threshold was applied to retain potentially ambiguous microbial reads, which were subsequently refined using alignment-based filtering.

A custom database was constructed with the parameters “--kmer-len 27 --minimizer-len 25 --minimizer-spaces 6”. The reference database included the human genome (GRCh37) to minimize misclassification of host-derived reads, along with bacterial, viral, and fungal reference genomes obtained from National Center for Biotechnology Information (https://www.ncbi.nlm.nih.gov, accessed on 22 November 2024). Reads classified as human were excluded prior to downstream microbial analysis.

Bracken [25] was used to estimate genus-level microbial abundances from Kraken2 classification outputs. While k-mer-based abundance estimation provides computational efficiency, its performance can be sensitive to database composition and read length, particularly in low-biomass samples.

To improve taxonomic specificity, a BLAST (ver. 2.2.31+) [26]-based approach was employed to refine taxonomic assignments of reads initially classified as microbial. This alignment-based strategy provides higher taxonomic specificity at the cost of increased computational complexity and was included to evaluate the impact of analytical stringency on downstream feature interpretation. An alignment length cutoff of 35 bp was applied to reduce spurious matches, and hits were filtered based on a hierarchical set of criteria including bit score, e-value, percentage identity, and alignment length to retain the most reliable taxonomic assignments. Due to the short-read length used in this study (38 bp), certain reads exhibited identical sequence matches to multiple genera within the same family, precluding unambiguous genus-level assignment. Such reads were therefore classified at the family level and are reported accordingly.

To further mitigate contamination effects in low-biomass cfDNA samples, taxa reported as potential contaminants in previous circulating microbiome studies [7,8,9,21] were used as a reference list. Among the taxa identified through feature selection, those overlapping with known contaminant lists were excluded from downstream analyses to reduce the influence of environmental and reagent-derived signals.

**Figure 2:**
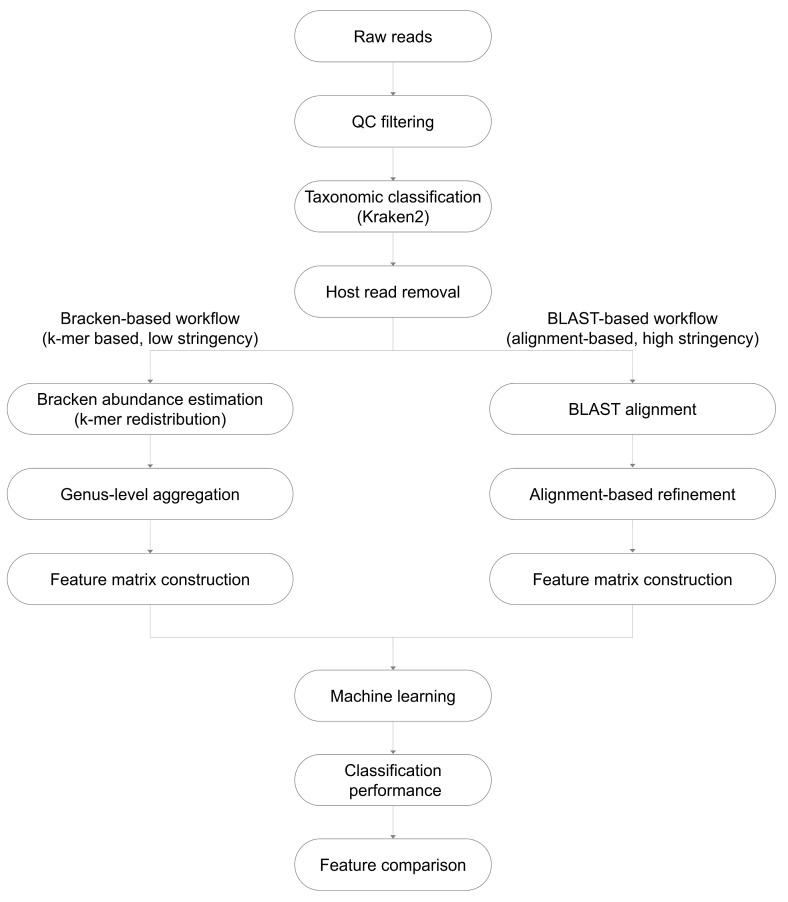
Schematic overview of the analytical workflow used for cfDNA-derived microbial profiling and downstream machine learning analysis. Raw sequencing reads undergo quality control (QC) filtering, followed by taxonomic classification using Kraken2 and host read removal. Two parallel taxonomic profiling strategies are then applied: a Bracken-based workflow (k-mer–based, low stringency), which performs abundance estimation through k-mer redistribution followed by genus-level aggregation, and a BLAST-based workflow (alignment-based, high stringency), which includes sequence alignment and alignment-based refinement. Both workflows generate feature matrices that are used for machine learning-based classification. Model performance is evaluated, and selected microbial features are compared between the two approaches.

Based on these approaches, two distinct taxonomic profiling workflows were implemented. In the Bracken-based workflow, Kraken2 classification outputs were directly used to estimate genus-level microbial abundances. In contrast, in the BLAST-based workflow, reads initially classified as microbial by Kraken2 were further refined using alignment-based taxonomic assignment to assess the impact of increased classification stringency. These workflows were applied in parallel to the same cfDNA dataset to enable direct comparison of pipeline-dependent effects. The overall decision framework for taxonomic assignment is illustrated in Figure 2.

### 2.4. Machine Learning Analysis

To mitigate the influence of contamination in low-biomass cfDNA data, taxa previously reported as common laboratory or reagent contaminants were excluded based on published studies [7,8,9,21]. To prevent potential bias in model evaluation, all preprocessing steps, including contaminant filtering and feature selection, were performed exclusively within each outer training fold, ensuring that no information from the held-out test fold influenced the feature set used for model training and evaluation.

Feature normalization was performed prior to model training and differed depending on the taxonomic profiling workflow. In the Bracken-based workflow, genus-level abundance values were scaled by multiplying by 10^6^. In the BLAST-based workflow, feature values were normalized by dividing fragment counts by the total number of assigned fragments per sample, resulting in relative abundance values.

A nested cross-validation framework (5-fold outer and 5-fold inner; performed once) was used. The inner loop was used exclusively for hyperparameter optimization, while model evaluation was conducted on held-out folds in the outer loop.

For each outer fold, the best hyperparameters identified from the inner cross-validation were used to refit the model on the entire outer training set. Feature selection was then performed within each outer training set by selecting the top 50 features based on feature importance derived from the refitted model, without involving the outer test fold. The number of selected features (top 50) was chosen to balance model interpretability and performance.

Models were then trained on the selected feature subsets within each outer training fold and evaluated on the corresponding held-out test fold. Hyperparameter optimization was performed using randomized search within the inner cross-validation loop.

Feature importance was defined using model-specific measures. For Random Forest (RF) [27], impurity-based feature importance values were used. For Logistic Regression (LR) [28], the absolute values of regression coefficients were used. For Multi-Layer Perceptron (MLP) [29], which does not inherently provide feature importance scores, Shapley additive explanations (SHAP) [30] values were used to estimate feature contributions.

For microbial profile-based lung cancer classification, MLP, LR, and RF models were implemented using the Scikit-learn library [31], representing nonlinear, linear, and tree-based classification approaches, respectively. All analyses were designed to minimize analytical bias in low-biomass cfmDNA studies.

## 3. Results

### 3.1. Microbial Taxonomic Profiling of cfDNA Samples

To characterize blood-derived microbial signals, taxonomic profiling of cfDNA sequencing data from 168 individuals was performed using two analytical approaches, Bracken-based and BLAST-based pipelines.

The Bracken-based approach identified 310 genera, whereas the BLAST-based approach identified 562 genera and 63 families. The increased number of taxa detected by the BLAST-based workflow likely reflects its alignment-based strategy, which enables more sensitive detection of low-abundance or ambiguously classified reads compared to k-mer-based classification.

A subset of taxa appeared to be consistently identified across both workflows, whereas many taxa were unique to each approach.

These differences indicate that the choice of taxonomic profiling method substantially influences both the number and composition of detected microbial features. While the BLAST-based workflow yielded a broader and more diverse set of candidate taxa, the Bracken-based workflow produced a more conservative feature set. This contrast highlights the impact of analytical stringency on microbial signal detection in low-biomass cfDNA data.

### 3.2. Classification Performance of Machine Learning Models

**Figure 3:**
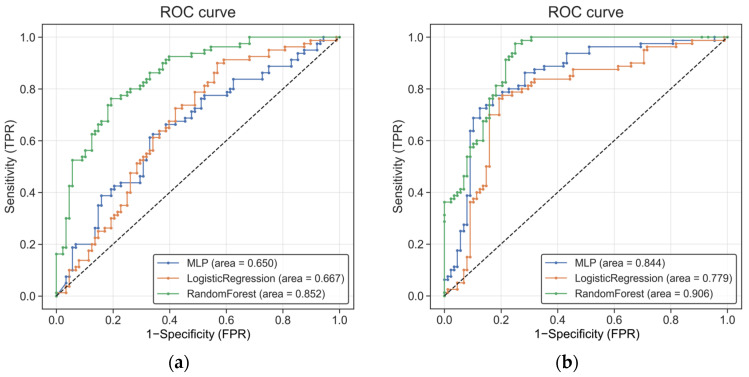
The receiver operating characteristic (ROC) curve of each approach: (**a**) ROC curves illustrating the diagnostic performance for lung cancer based on a machine learning model trained on Bracken-derived data; (**b**) ROC curves illustrating the diagnostic performance for lung cancer based on a model trained on BLAST-derived data. In both panels, the x-axis represents the false positive rate (FPR) and the y-axis represents the true positive rate (TPR). The area under the curve (AUC) indicates overall diagnostic performance. Multi-Layer Perceptron (MLP), Logistic Regression (LR), and Random Forest (RF) are represented by blue, orange, and green lines, respectively. The black dashed line represents the performance of a random classifier (AUC = 0.5), serving as a baseline for comparison.

We next evaluated whether microbial profiles derived from each taxonomic profiling strategy could discriminate lung cancer patients from non-cancer controls using supervised machine learning models. Overall model performance is summarized in Table 2 and illustrated by receiver operating characteristic (ROC) curves in Figure 3.

**Figure 4:**
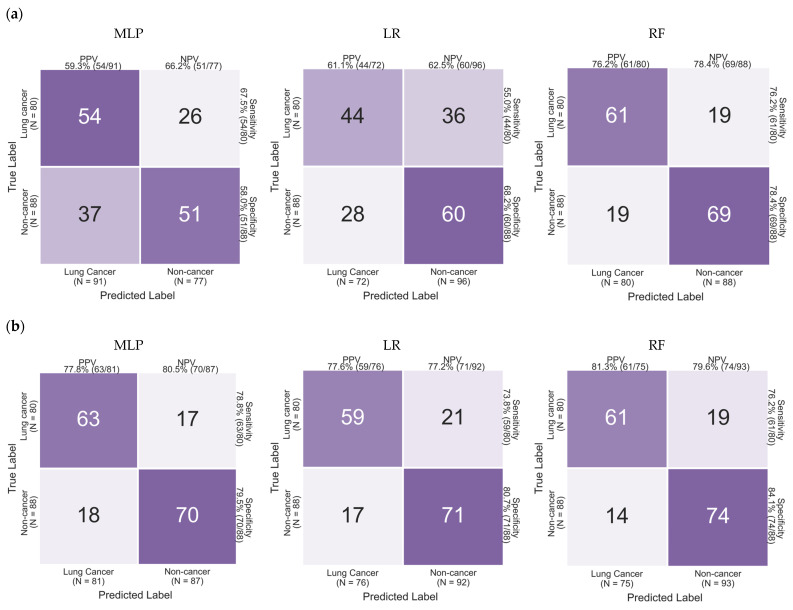
Confusion matrices of machine learning models for lung cancer classification: (**a**) Confusion matrices derived from Bracken-based features using Multi-Layer Perceptron (MLP), Logistic Regression (LR), and Random Forest (RF); (**b**) Confusion matrices derived from BLAST-based features using MLP, LR, and RF. True labels are shown on the y-axis and predicted labels on the x-axis. Sensitivity, specificity, positive predictive value (PPV), and negative predictive value (NPV) are indicated for each model.

For models trained on Bracken-derived profiles, classification performance varied substantially across algorithms. The Random Forest (RF) model achieved the strongest discrimination, with an area under the curve (AUC) of 0.852, sensitivity of 76.2%, and specificity of 78.4% (Figure 3). In contrast, Logistic Regression (LR) and Multi-Layer Perceptron (MLP) models demonstrated comparatively modest performance (AUC 0.667 and 0.650, respectively), suggesting limited linear separability of microbial features under this profiling strategy. Detailed confusion matrices are provided in Figure 4.

When trained on BLAST-refined microbial profiles, all classifiers showed improved discrimination relative to their Bracken-based counterparts. The RF model achieved the highest overall performance (AUC 0.906, sensitivity 76.2%, specificity 84.1%), while MLP (AUC 0.844) and LR (AUC 0.779) also demonstrated notable performance gains. These improvements suggest that alignment-based refinement may enhance the signal-to-noise ratio of microbial features, particularly in low-biomass cfDNA data.

Across both taxonomic profiling strategies, the RF classifier consistently achieved the highest performance, while linear models showed comparatively lower discrimination. This pattern indicates that nonlinear interactions among microbial features contribute to classification performance.

Importantly, although the BLAST-based workflow yielded higher classification performance, the magnitude of improvement varied across models, indicating that model performance is influenced not only by taxonomic profiling strategy but also by the interaction between feature representation and model architecture.

These findings suggest that lung cancer classification may depend on combined microbial patterns across multiple taxa rather than on single discriminative features. Overall, the observed differences in performance highlight that analytical choices in taxonomic profiling can meaningfully influence downstream model behavior and interpretation.

### 3.3. Feature Importance Patterns Across Profiling Strategies

To investigate how individual microbial genera contributed to lung cancer classification, we analyzed feature importance scores derived from the Random Forest (RF) model for each taxonomic profiling strategy. Because the RF classifier consistently achieved the highest classification performance across both Bracken- and BLAST-derived datasets, RF-based importance measures were used as the primary criterion for feature ranking to ensure methodological consistency and robustness.

Within each outer cross-validation fold, microbial genera were ranked according to their RF-derived importance scores, and the top 50 genera were selected for downstream comparison. This threshold was chosen to balance feature stability, model complexity, and interpretability.

Although classification performance was robust under both profiling workflows, the distribution and ranking of highly important genera differed substantially between the Bracken- and BLAST-derived datasets. While a subset of genera was consistently identified across both workflows, a considerable proportion of features were unique to each approach, indicating that feature attribution is sensitive to the choice of taxonomic profiling strategy.

These findings suggest that differences in taxonomic assignment not only affect feature detection but also influence the relative importance of microbial taxa in classification models. This variability highlights the potential for pipeline-dependent bias in interpreting microbiome-derived features from cfDNA data.

To further characterize these patterns, genera consistently selected across workflows were examined in detail in the following section.

### 3.4. Pipeline-Dependent Feature Selection Across Cross-Validation Folds

To evaluate the stability of discriminative taxa, we examined the frequency and overlap of genera selected among the top 50 RF-based features across the five cross-validation folds for each profiling strategy.

A total of 87 genera were selected at least once in the Bracken-derived data, whereas 65 genera were selected in the BLAST-derived data. Of these, 38 genera overlapped between the two approaches, indicating partial reproducibility but substantial pipeline-dependent variation in feature selection.

Importantly, 13 genera (*Acinetobacter*, *Aeromonas*, *Cloacibacterium*, *Cutibacterium*, *Diaphorobacter*, *Ectopseudomonas*, *Escherichia*, *Flavobacterium*, *Janthinobacterium*, *Klebsiella, Pseudomonas*, *Sphingobium*, *Xanthomonas*) were consistently selected among the top 50 features across all five cross-validation folds in both workflows. These genera represent the most robust and reproducible microbial signals identified in this study. The relative abundance patterns of these consistently selected genera differed between the two workflows, with some taxa showing concordant trends while others exhibited workflow-dependent variation (Supplementary Table S1 (Sheet 2)).

In addition to the shared features, each profiling strategy identified distinct sets of consistently selected taxa. Among the Bracken-derived features, 49 genera were unique to this approach, including 9 that were selected in all five folds. Conversely, 27 genera were uniquely identified in the BLAST-based analysis, with 13 consistently selected across all folds.

These findings indicate that while a core subset of microbial features can be reproducibly identified, a substantial proportion of discriminative taxa remains sensitive to the choice of taxonomic profiling strategy. This highlights the importance of analytical consistency when interpreting microbiome-derived biomarkers from cfDNA data.

### 3.5. Inconsistency of Fold Change Patterns Among Selected Genera

Having identified genera consistently selected as discriminative features, we next examined whether these taxa exhibited consistent abundance patterns between lung cancer and non-cancer samples. Notably, consistent feature selection does not necessarily imply concordant directionality of association.

Fold change (FC) values were calculated separately for Bracken- and BLAST-derived datasets as the ratio of the average read count in lung cancer samples to that in non-cancer controls.

Among the shared genera, *Acidovorax* showed modestly higher abundance in lung cancer samples in both datasets (FC > 1 in both approaches), whereas *Acinetobacter* exhibited lower relative abundance in lung cancer samples across methods (FC < 1) (Supplementary Figure S1 and Table S1).

In contrast, several genera demonstrated method-dependent differences in quantitative trends. For example, *Klebsiella* and *Pseudomonas* were consistently selected across cross-validation folds in both pipelines; however, their FC values differed in direction between Bracken- and BLAST-derived data. These discrepancies indicate that reproducible feature selection does not necessarily correspond to a consistent biological interpretation.

Additionally, several genera uniquely identified in the Bracken-derived workflow exhibited reduced abundance in lung cancer samples but were not consistently detected in the BLAST-based analysis.

Together, these findings demonstrate that taxonomic profiling strategy can influence not only feature selection but also the inferred direction of association between microbial taxa and disease status. This highlights a critical challenge in cfDNA-based microbiome analysis, where analytical variability may directly impact biological interpretation and clinical translation.

## 4. Discussion

In this study, we evaluated how taxonomic profiling strategies influence machine learning-based classification and microbial feature interpretation in cfDNA-derived blood microbiome analysis using two analytical workflows: a Bracken-based abundance estimation approach and a BLAST-refined alignment-based strategy. While classification performance remained robust, feature-level interpretation showed substantial sensitivity to the chosen bioinformatics pipeline.

A key finding of this study is that comparable classification performance does not necessarily translate into consistent biological interpretation. Although a subset of microbial genera was consistently selected across cross-validation folds and profiling strategies, quantitative FC patterns were not uniformly aligned between methods. In several cases, taxa reproducibly identified as important features exhibited method-dependent differences in inferred abundance trends. These results highlight that analytical design choices can meaningfully influence feature attribution, even when overall discrimination metrics appear stable.

Consistent with the superior performance of the RF classifier, feature importance analyses were conducted using RF-derived importance measures to ensure methodological consistency across profiling strategies. The consistent advantage of RF across both workflows suggests that lung cancer classification is driven by combined microbial patterns across multiple taxa rather than single dominant features.

Importantly, stability in feature selection did not uniformly translate into concordant abundance directionality. Even genera consistently identified as important features demonstrated variability in inferred FC patterns between Bracken- and BLAST-derived datasets. In low-biomass cfmDNA analyses, such discrepancies may arise from differences in taxonomic assignment stringency, database composition, read filtering criteria, and alignment strategies. These findings underscore the need for cautious biological interpretation when attributing disease relevance to individual taxa.

Several genera identified in this study have been reported in previous lung cancer-associated microbiome studies [9,10,32,33]. However, prior reports have shown variability in both taxonomic composition and directionality of association. Our findings suggest that part of this heterogeneity may stem from differences in taxonomic classification strategies and abundance estimation frameworks. In low-biomass blood microbiome analyses, where microbial signals are inherently weak and susceptible to contamination and technical noise, bioinformatics pipeline choices may exert a disproportionate influence on inferred microbial patterns.

An additional consideration in low-biomass cfDNA microbiome studies is the potential impact of environmental and reagent contamination. Because circulating microbial DNA is present at extremely low abundance, even minor background contamination may disproportionately influence taxonomic profiles and downstream feature attribution [7,8,21]. In this study, no-template or reagent-only negative controls were not included, limiting the ability to experimentally distinguish biological signal from background contamination. This reflects the retrospective nature of the dataset, in which experimental controls could not be incorporated.

To partially address this concern, genera previously reported as common laboratory or reagent contaminants [7,8,21] were excluded during feature selection based on published contamination studies, and this filtering was applied independently within each cross-validation fold to minimize potential bias in model evaluation. Nevertheless, computational mitigation strategies cannot fully substitute for experimental controls, and residual contamination effects cannot be completely excluded. Future studies incorporating reagent-only negative controls and spike-in standards will be essential for confirming biological signal robustness.

This study has several limitations. First, experimental negative controls were not included. Second, sequencing depth was relatively shallow, potentially amplifying sensitivity to analytical variability. These limitations are particularly important in low-biomass cfDNA microbiome studies, where signal-to-noise ratios are inherently low. In addition, fourteen samples were excluded due to sequencing quality issues, which may introduce potential selection bias. Third, external validation in independent cohorts was not performed. Additionally, individual-level age information and detailed comorbidity data were not available in the clinical records used for this study and could not be included in the patient characteristics table. These factors may influence microbial profiles and potentially confound classification results, representing an additional limitation of the present study. Future studies incorporating contamination-aware experimental controls, deeper sequencing, standardized analytical frameworks, external validation, and more comprehensive clinical metadata will be necessary to rigorously evaluate the robustness and reproducibility of cfDNA-derived microbial signatures.

In a clinical diagnostic setting, analytical reproducibility is as critical as classification accuracy. Our findings highlight that standardized and reproducible bioinformatics pipelines are a prerequisite for the reliable clinical application of cfDNA-derived microbial biomarkers.

## 5. Conclusions

cfDNA-based blood microbiome profiling can support machine learning-based discrimination of lung cancer status while remaining highly sensitive to analytical design choices. Our findings underscore the importance of pipeline-aware interpretation and caution against overreliance on individual taxa as definitive disease biomarkers. By explicitly examining method-dependent variability, this study contributes to a more rigorous framework for low-biomass cfmDNA research. These findings emphasize that consensus-driven and standardized bioinformatics frameworks are essential for the safe, reproducible, and clinically reliable application of cfmDNA profiling in liquid biopsy diagnostics.

## Figures and Tables

**Table 1:** Patient characteristics.

Characteristics		Normal	Lung Cancer
Enrollment		88	80
Gender	Male	30 (34.1%)	48 (60.0%)
	Female	58 (65.9%)	32 (40.0%)
Stage	I	- ^1^	26 (32.5%)
	II	-	14 (17.5%)
	III	-	34 (42.5%)
	IV	-	5 (6.3%)
	Not specified	-	1 (1.2%)
Cancer Type	Adenocarcinoma	-	47 (58.8%)
	Squamous cell carcinoma	-	16 (20.0%)
	Others	-	17 (21.2%)

^1^ ‘-’ indicates not applicable, as cancer staging is not defined for normal samples.

**Table 2:** Total 5-fold cross-validation performance evaluation of each method.

Approach	Model	AUC	Sensitivity	Specificity	PPV	NPV
Bracken	MLP	0.650	67.5%	58.0%	59.3%	66.2%
	LR	0.667	55.0%	68.2%	61.1%	62.5%
	RF	0.852	76.2%	78.4%	76.2%	78.4%
BLAST	MLP	0.844	78.8%	79.5%	77.8%	80.5%
	LR	0.779	73.8%	80.7%	77.6%	77.2%
	RF	0.906	76.2%	84.1%	81.3%	79.6%

## Data Availability

The datasets used and/or analyzed during the current study are available from the corresponding author upon reasonable request.

## Supplementary Materials

The following supporting information can be downloaded at: https://www.mdpi.com/article/10.3390/diagnostics16071079/s1, Figure S1: Fold change values of selected bacterial genera derived from Bracken- and BLAST-based analyses; Table S1: Frequency of genus selection among the top 50 most important features across five cross-validation folds for Bracken- and BLAST-derived datasets.

## Abbreviations

The following abbreviations are used in this manuscript:

cfDNA	circulating cell-free DNA
cfmDNA	circulating cell-free microbial DNA
AUC	Area Under the Curve
RF	Random Forest
MLP	Multi-Layer Perceptron
LR	Logistic Regression
FC	Fold change
GRCh37	Genome Reference Consortium human genome build 37
